# Prediction of food allergy reaction severity: biomarkers and host factors

**DOI:** 10.3389/falgy.2025.1578601

**Published:** 2025-08-13

**Authors:** David J. Fitzhugh

**Affiliations:** Allergy Partners of Chapel Hill, Chapel Hill, NC, United States

**Keywords:** anaphylaxis, basophil activating test (BAT), food allergy severity, biomarkers, component resolved allergy diagnostics

## Abstract

Prediction of food allergy reaction severity remains a challenging clinical dilemma, with no single biomarker or patient factor serving as a definitive predictor. Clinically, being able to accurately estimate future reaction severity would be a key advancement in terms of risk-stratifying patients who might most benefit from specific immunotherapy, anti-IgE therapy, or at minimum, ensuring this population always has autoinjectable epinephrine. This mini-review explores advancements in two key domains: biomarkers and host factors. Biomarker studies highlight the predictive limitations of IgE sensitization levels, while emerging tools such as basophil activation tests (BAT) and bead-based epitope assays (BBEA) are promising but are not yet in widespread use. Specifically, BAT demonstrates superior discriminatory power for severe peanut and baked egg reactions, whereas Arah2 component level above 1.4 kU/L suggest a more severe peanut allergy phenotype. Host factors, including comorbid conditions, age, and behavioral variables, further complicate severity prediction. While asthma has frequently been assumed to be involved in more severe reactions, recent meta-analyses refute this association unless asthma is poorly controlled. Similarly, a history of anaphylaxis does not reliably predict future reaction severity. Age emerges as a significant variable, with adolescents through the fourth decade of life displaying a higher risk for severe reactions. Additionally, cofactors such as exercise, alcohol, and certain medications may modulate reaction severity, albeit with varying degrees of evidence. Despite these advances, significant knowledge gaps remain in predicting reaction severity with high confidence. The future likely lies in a multifactorial approach. Understanding the interplay of biomarkers and host factors will be crucial in developing more accurate predictive models, ultimately enhancing food allergy management and patient safety.

## Introduction

This mini-review will focus on food allergy reaction severity from two perspectives: advances in biomarkers and host factors. The latter can encompass comorbid disease states such as asthma, non-modifiable factors such as age, and modifiable factors such as risk-taking behavior and exercise. Identifying both objective biomarker trends and patient factors that can accurately predict reaction severity remains an elusive challenge and it should be acknowledged from the outset that we are far from achieving this goal but it is worth reviewing the progress to date and how the available knowledge can be leveraged to help with such predictions, as well as dispelling some commonly-held notions that are not accurate.

### Biomarkers

#### Degree of IgE sensitization/component resolved diagnostics

Most allergists are comfortable with the concept that the degree of IgE sensitization, whether to whole allergen or specific allergen component, is more predictive of likelihood of reaction rather than severity of reaction. Numerous studies confirm that the severity of a given reaction is not correlated to specific IgE level (1). Initial studies of Arah2, the most well-characterized biomarker for systemic reactions to peanut, demonstrated that the magnitude of Arah2 positivity is not associated with reaction severity or an ability to predict anaphylaxis (2). It is important, however, to distinguish the potential for any systemic reaction from the potential for anaphylaxis: there is an association with higher levels of Arah2 (peanut), Jug r1 (walnut), and Cor a 14 (hazelnut) predicting a greater likelihood of any systemic reaction (1).

One area where component markers can be quite helpful is distinguishing pollen-food allergy syndrome (PFAS) from the potential for more severe reactions. Sensitization to Ara h8 (the Bet v1 homolog in peanut) is clearly associated with a milder reaction phenotype, typically associated with PFAS (3). Similarly, sensitization exclusively to Cor a1 (the Bet v1 homolog in hazelnut) is associated with a milder PFAS phenotype (4). While many allergists are familiar with component-resolved diagnostics, it's important to understand their strengths and limitations. A clear strength is the ability to generally distinguish a pure PFAS phenotype (such as mono-sensitization to Ara h8 or Cor a1). However, an obvious limitation is that the presence of sensitization to seed storage proteins (such as Ara h2 or Cor a14) does not automatically inform of potentially severe reactions (though they do imply some increased potential for systemic reaction). However, relatively recent reports from Santos et al, using the LEAP and LEAP-On cohorts, report a 100% sensitivity and 93% specificity for severe peanut reactions using an Ara h2 cutpoint of 1.4 kU/L (5). Thus, at least for Ara h2 in peanut allergy, there is accumulating evidence of association with reaction severity.

#### Basophil activation test/mast cell activation test

Basophil activation testing is increasingly available in clinical practice, though it remains a fairly specialized assay and as a live cell assay, requires both timeliness of the run and expertise with both technical aspects as well as interpretation. That said, the BAT is emerging as perhaps the most compelling tool for prediction of reaction severity. Basophil activation testing can be thought of as, in effect, an *in vitro* challenge: flow cytometry is used to detect cell surface marker changes with increasing allergen concentration, with both the percentage of responder cells and the threshold of response being quantified (6). In the analysis by Santos et al. of the LEAP and LEAP-On cohorts, BAT had the best discriminatory ability for prediction of severe or life-threatening reactions to peanut, with a sensitivity of 100% and a specificity of 97% (5). In a separate report, Cottel et al. propose that using a multivariate model of Ara h2 level and FceR1-positive control BAT values led to a 92% sensitivity and 82% specificity for severe peanut reactions (7). It should be noted that BAT FcER1 positivity is an allergen-independent marker and may be correlated to basophil activation potential in food allergy in a manner analogous to the correlation to increased serum tryptase levels in patients with severe venom allergy (7). A recent report from Radulovic et al. in a study of the performance of ovomucoid sp IgE vs. BAT for prediction of severe reactions to baked egg revealed that BAT offered the best diagnostic accuracy at 75%, with a sensitivity of 76% percent and a specificity of 74% at optimal CD63% positive cut-off, whereas ovomucoid sp IgE offered only 60% accuracy at optimal cut-off (8). Finally, while reaction threshold and reaction severity have typically been considered independent of each other, Santos et al. note a strong correlation between *in vitro* BAT markers of severity and threshold, which supports at least an indirect relationship between low threshold to react and increasing reaction severity (5).

Mast cell activation testing (MAT) potentially overcomes two of the primary limitations of the BAT, namely that about 10%–15% of BAT subjects are positive control non-responders and that BAT can only be performed on live patient sample cells, which limits usage to (at most) 24 h after obtaining the specimen (9). While MAT shares many conceptual similarities to BAT (using flow cytometry to monitor activation markers after *in vitro* allergen stimulation), there are several key differences. First, MAT uses either an immortalized mast cell line or primary human mast cells from healthy donors, neither of which is patient-specific. Second, MAT requires passive sensitization of either mast cell source with patient IgE, which lowers the sensitivity of MAT vs. BAT (75% vs. 83% in one report) (10). Thus, while not dependent on live cells from the allergic subject (which eliminates the timeliness concern of BAT), the ultimate lower sensitivity of MAT suggests that it may be better positioned as a fall-back test for BAT non-responders (11). Moreover, MAT is far less available than BAT (which itself is not universally available at this time).

#### Bead-based epitope assay (BBEA)

The bead-based epitope assay represents a further refinement of component-resolved diagnostics, in effect allowing a high-throughput assessment of sensitization to specific linear allergen epitopes. This assay involves mechanically coupling peptides which represent epitopes of interest to microbeads, incubation with patient serum, followed by multiplex analysis of fluorophore-labelled secondary antibodies (10). In the US, this assay has been commercialized for peanut, with a combination of epitopes Ara h2_008 and Ara h2_0019 yielding a sensitivity of 92% and a specificity of 94% for peanut allergy (6). Moreover, by correlating the epitope “maps” of patients with well-characterized food challenge cohorts, this assay aims to predict threshold dose of reaction in three strata (low, medium, or high tolerance) (12). There has been some suggestion that a higher diversity of recognized epitopes could correlate to increased reaction severity but the current commercial assay for peanut does not attempt to predict reaction severity (13). An important limitation of the BBEA is that it can only assess linear/sequential epitopes, whereas conformational epitopes (related to the folding pattern of the protein) cannot be assessed by BBEA but could play an important role in reaction severity (10).

### Host factors

#### Asthma

It is a common assumption that a history of asthma is associated with the potential for more severe food allergy reactions (14). However, notably, a history of asthma alone is not a predictor for severe or fatal food anaphylaxis (15). Turner et al. performed a meta analysis of 32 primary research studies evaluating the relationship between asthma and reaction severity and concluded that there was “no consistent evidence that asthma is associated with increased severity of food-induced reactions or the need for ICU admission and/or intubation and mechanical ventilation” (1). Thus, while respiratory compromise is the most typical manifestation of a life-threatening food reaction, asthma status alone does not increase the risk of severe reaction. That said, it remains an important clinical goal to achieve optimal control of asthma for all asthmatic patients, both with and without concomitant food allergy.

#### Allergic rhinitis and eczema

In a recent UK study, there was a seasonally higher rate of hospital admission for food anaphylaxis during the tree pollen season, though this did not extend to fatal food anaphylaxis (16). Conversely, Turner et al. note that this seasonal phenomenon has not been observed for ICU admissions due to food allergy in North America and speculate that since the baseline risk for fatal and near-fatal food reactions is so minimal, “any impact of concomitant atopic disease is negligible” (1). Regarding eczema, there does not seem to be any significant association with food allergy reaction severity and eczema, despite atopic dermatitis being a well-known harbinger of food allergy in affected infants and toddlers (1, 17).

#### Mastocytosis and hereditary alpha tryptasemia

While it has been well-established that there is a clear association between severe hymenoptera reactions and elevated baseline tryptase (whether due to clonal mast cell disorders or alpha tryptasemia), this relationship does not hold true with food allergy (1). Dua et al. did not report any association between baseline tryptase and food allergy reaction severity, though perhaps unsurprisingly, they did observe a clear correlation between percent tryptase rise over baseline tryptase and reaction severity (18). Thus, while interesting to have dynamic tryptase data during the course of a reaction, there would not seem to be value in drawing routine baseline tryptase levels for food allergy patients unless there is otherwise independent suspicion of a mast cell disorder.

#### Past history of anaphylaxis

It is a frequent assumption by parents of food allergic children that a prior history of anaphylaxis portends future severe reactions as well as the concept that every subsequent exposure will result in a progressively more severe reaction. However, Turner et al. reinforce from multiple studies that “a history (or not) of anaphylaxis is a poor predictor of future anaphylaxis.” (1, 19) Thus, it is important to reinforce to parents and patients that while a mild past reaction does not preclude a severe future reaction, one likewise cannot infer that a severe prior reaction is a harbinger of future reaction intensity. Most importantly, we need to reinforce vigilance in the carrying of epinephrine auto-injectors for food allergy patients and not to presume that a history of only mild reactions is a rationale to avoid carrying emergency medications.

#### Threshold dose/severity relationship

In a prospective DBPCFC trial of children with milk, egg, peanut, cashew, and/or hazelnut allergy, there was minimal relationship between eliciting dose and ultimate reaction severity. The authors note that “clinicians should not make decisions regarding prescription of epinephrine auto-injectors or give advice about the level of stringency of allergen avoidance based on the eliciting dose obtained from graded food challenges, as eliciting dose only contributes marginally to reaction severity.” (19). Likewise, Turner et al. conclude that there is no compelling evidence of a relationship between very low eliciting dose reactors and more severe reactions (1).

#### Age/risk-taking behavior

While anaphylaxis is most commonly reported in infants and toddlers, adolescents and adults are actually at greatest risk of fatal and near-fatal anaphylaxis (1, 20). It has also sometimes been (incorrectly) assumed that risk-taking behaviors among adolescents and young adults with food allergy might lead to more severe reactions, but Turner et al. noted that the age-related increase in fatal and near-fatal anaphylaxis was maintained well into the fourth decade of life, a point at which time one presumes purposeful risk-taking behaviors vis-a-vis food allergy would likely be decreased with maturity (21). Thus, age alone (even beyond the “risk-taking” adolescent years) does appear to be a significant factor in more severe reactions.

#### Exercise

Exercise is one of the best described augmenting cofactors in reaction severity, both in the context of food-dependent, exercise induced anaphylaxis (FDEIA), as well as a commonly cited cofactor in severe reactions among patients on oral immunotherapy (OIT). Classically, FDEIA has been thought of as a food-associated reaction occurring only in the context of significant exertion, though a report from Christensen et al. noted that among a cohort of adults with confirmed wheat-associated FDEIA, 26 of 71 actually reacted at very high doses of wheat even absent exercise (22). Thus, the notion of “pure” FDEIA may be misinformed and exercise may in effect serve to both lower the threshold and increase the severity of some food reactions, even though many individuals tolerate typical doses with no ill effects absent exercise.

#### Alcohol and medications

While alcohol has been cited as a possible factor in up to 16% of accidental exposure reactions in a food-allergic adult population, the presence of alcohol as a cofactor did not seem to increase reaction severity in this study (23). That said, alcohol consumption could clearly increase risk-taking behavior and alcohol is known to be a direct mast cell agonist, so even if there is no effect on severity, both frequency of reactions and threshold of reaction might be impacted by consuming alcohol (24).

Regarding both beta-blockers and ACE inhibitors, these medications have been linked in a recent meta-analysis to potentially more severe anaphylaxis outcomes, but the quality of evidence was low due to presence of confounding cardiovascular disease in this patient population (25). Of note, in a case study of food reactions resulting from accidental exposure, prescriptions for either beta blockers or ACE inhibitors were not present in higher frequency in patients who had reactions categorized as severe (23).

#### Viral infection

Concomitant viral infection during oral immunotherapy is a frequently cited explanation for possible reactions during OIT, with the presumed mechanism being a transient lowering of reaction threshold to a previously tolerated dose (26). However, in a study of 157 adults with food allergy, there was no relationship noted between infection and accidental exposure reaction severity over a one year observation period (23).

### Limitations of this review

This mini-review is exclusively focused on two aspects of food allergy reaction severity, biomarkers and patient factors, and is not meant to be comprehensive. There are certainly other factors that impact reaction severity, such as intrinsic allergen factors, food matrix, and allergen processing, but those factors are beyond the scope of this brief review.

## Discussion

Prediction of the severity of food allergy reactions is a significant knowledge gap in the food allergy space and while there have been advances, there remains no single biomarker or patient factor that is a singular predictor of severity. However, there have been advances, particularly in the biomarker arena: Arah2 for peanut at levels above 1.4 kU/L suggests a more severe reaction phenotype and the basophil activation test holds promise for predicting reaction severity to a variety of allergens, with published data for both peanut and baked egg as detailed in this report. Of the various patient factors, age (adolescence through the fourth decade of life) is notably the most highly associated variable associated with reaction severity.

It is also important to dispel two long-held notions that both patients and clinicians have frequently associated with reaction severity. First, a history of asthma alone is not correlated with severe food allergy reactions (though poorly controlled asthma may be). Second, a past history of severe reaction doesn’t portend future severe reactions or refractory anaphylaxis.

As the state of the art continues to advance, allergists will have more precision tools available to help guide prediction of food allergy reaction severity. However, it is unlikely that there will be one single biomarker that can be absolutely predictive. Most likely, allergists will need to embrace both new laboratory assays (particularly BAT and MAT as the most physiologic challenge surrogates) in conjunction with patient risk factors to provide the most accurate prediction of food allergy reaction severity.
